# Emerging imaging modalities for the diagnosis of malignant lesions of the male reproductive tract in companion animals

**DOI:** 10.1590/1984-3143-AR2025-0124

**Published:** 2026-04-10

**Authors:** Diego Rodrigues Gomes, Anna Carolina Mazeto Ercolin, Gabriela Castro Lopes Evangelista, Felipe Farias Pereira da Câmara Barros, Camila Silveira Stanquini, Camila Debastiani, Fredderico Garcia, Marcus Antônio Rossi Feliciano

**Affiliations:** 1 Departamento de Medicina Veterinária, Faculdade de Zootecnia e Engenharia de Alimentos – FZEA, Universidade de São Paulo – USP, Pirassununga, SP, Brasil; 2 Departamento de Medicina e Cirurgia Veterinária, Instituto de Veterinária, Universidade Federal Rural do Rio de Janeiro – UFRRJ, Seropédica, RJ, Brasil; 3 Empresa Conectus, Jundiaí, SP, Brasil

**Keywords:** radiography, reproduction, small animals, tumors, ultrasound

## Abstract

Understanding diseases that affect the male reproductive system of dogs and cats is important for improving their health and quality of life. Imaging techniques, such as ultrasound and X-rays, play a fundamental role in the evaluation of this system. They help veterinarians detect problems in the testicles, prostate, and other reproductive organs of dogs and cats. In recent years, advanced ultrasound methods, such as Doppler, elastography, and contrast-enhanced ultrasound, have become more accessible and allow earlier and more accurate diagnosis. Although definitive diagnosis of neoplastic lesions depends on cytological or histopathological examinations, radiography and advanced ultrasonography contribute significantly to early detection and malignancy characterization. This literature review explores the main imaging techniques used in the investigation of malignant lesions in the male reproductive tract of dogs and cats, namely radiography, B-mode and Doppler ultrasonography, elastography, and contrast-enhanced ultrasonography (CEUS). The goal is to help veterinarians make better decisions and improve care for their patients. The integration of these modalities has become well established in human medicine and shows growing applicability in veterinary practice, allowing for real-time, non-invasive assessment of the testes, prostate, penis, and scrotal structures. The compiled data highlight the potential of combining different imaging modalities to improve diagnostic accuracy and provide clinical and surgical support in the management of malignant lesions affecting the male reproductive tract in dogs and cats.

## Introduction

The male reproductive system of dogs and cats comprises the organs involved in the production, transport, and storage of male gametes (spermatozoa), including the scrotum, testes, epididymides, spermatic cords, penis, urethra, and accessory sex glands (prostate and bulbourethral glands) (Konig and Leibich, 2016).

Lesions of the male reproductive tract are frequently reported in dogs (Foster, 2012). The testis is the second most common site for tumor development in male dogs. In contrast, prostatic tumors are uncommon in this species. Testicular and prostatic tumors are rarely reported in cats (McEntee, 2002). Urethral and penile neoplasms are relatively rare, with transmissible venereal tumors (TVTs) and squamous cell carcinomas being the most common neoplasms in male dogs at these sites (Hall et al., 1976; McEntee, 2002). Imaging findings related to the evaluation of the spermatic cord are scarce in the literature (Mantziaras, 2020).

Diagnostic imaging is recognized as one of the most important tools for assessing the reproductive tract in small animals (Mantziaras, 2020). This review aims to describe the use of radiography and ultrasonography, as well as advanced imaging modalities, as diagnostic tools for detecting malignancy in lesions of the male reproductive system in dogs and cats.

## Applicability of radiology

Despite advances in various imaging modalities, radiography remains a widely used technique due to its low cost, rapid execution, and availability in veterinary facilities, in addition to its relatively simpler interpretation, especially when compared to ultrasonography (Meomartino et al., 2021).

**Figure 1:**
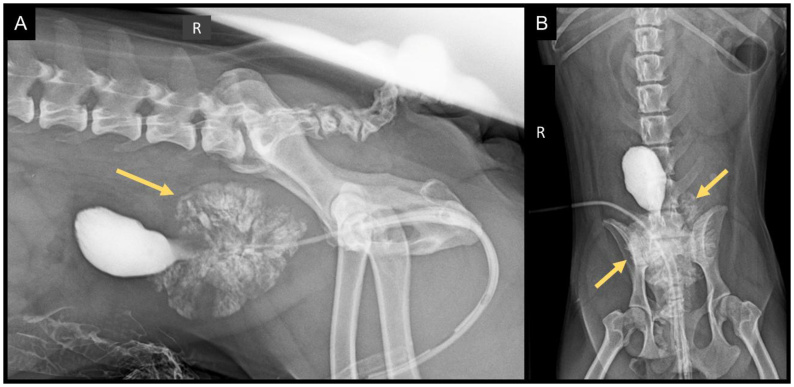
Contrast radiography in lateral (A) and ventrodorsal (B) projections revealing prostatitis and paraprosthetic abscess. The prostate is located intra-abdominally, exhibiting increased dimensions and irregular contour demonstrated by contrast extravasation from the prostatic urethra (arrows). Image kindly provided by Douglas Mattei and Letícia Pina, CEMEV.

Radiography can be used to assess the size, shape, contour, and location of the prostate in dogs (Atalan et al., 1999). In general, the most common radiographic finding associated with prostatic disorders in dogs is organ enlargement (Johnston et al., 1991). However, radiographically visualized lesions are nonspecific, and it is not possible to determine whether the observed changes are due to hyperplasia, infection, or neoplasia (Atalan et al., 1999). Prostatic enlargement may present as either symmetrical or asymmetrical. Inflammatory diseases/prostatitis (Figure 1) and hypertrophy typically result in symmetrical, circumferential enlargement, whereas asymmetrical enlargement is usually associated with cysts, abscesses, or neoplasms (Johnston et al., 1991).

In the ventrodorsal projection, the normal prostate should not exceed 50% of the width of the pelvic inlet. In lateral projections, the proportion occupied by the gland should not exceed 70% of the distance between the sacral promontory and the pubic bone. In cases of marked prostatomegaly, occupying more than 90% of the sacropubic distance, neoplasia, abscess, or prostatic cyst should be considered as possible differential diagnoses (Feeney et al., 1987). Radiographic assessment of prostatic length may be as useful as ultrasonographic measurement, as a strong correlation has been observed between the lengths obtained by both methods (Johnston et al., 1991).

The evaluation of prostatic margins can significantly contribute to the differentiation among various conditions. Poorly defined or irregular margins are frequently observed in cases of neoplasia or abscesses, reflecting aggressive and infiltrative processes. In contrast, well defined and smooth margins are common features of benign prostatic hyperplasia (BPH) and other slowly progressing, non-invasive conditions. More aggressive processes, whether neoplastic or infectious, may invade or penetrate the prostatic capsule and trigger an inflammatory response in the adjacent periprostatic tissues (Johnston et al., 1991).

**Figure 2:**
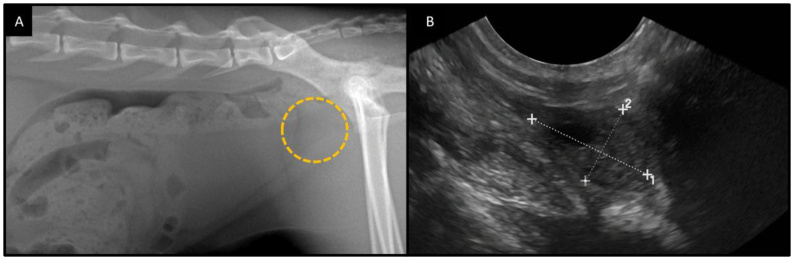
Prostatic carcinoma in the cat. (A) Radiograph in the lateral view showing the cranial displacement of the urinary bladder possibly associated with a prostatic enlargement (dashed line); (B) Ultrasonography of the enlarged prostate in longitudinal view (calipers).

Although radiography is not the most commonly used technique currently for the evaluation of prostatic diseases, it complements the diagnostic investigation regarding the topography, enlargement, or calcification of the prostate, as well as involvement of adjacent bony structures (vertebral bodies), lungs, and regional lymph nodes (iliac) due to metastatic neoplastic processes (Smith, 2008; Lévy et al., 2014) (Figure 2). Variations in the radiographic density of the prostate are uncommon. Punctate mineralizations are associated with benign prostatic concretions, abscesses, or neoplasms. In castrated dogs, prostatic mineralization is highly associated with neoplasia, with a positive predictive value of 100%, negative predictive value of 50%, specificity of 100%, and sensitivity of 84% (Bradbury et al., 2009). In intact animals, prostatic mineralizations may or may not be associated with malignant processes, showing a positive predictive value of 22%, negative predictive value of 96%, sensitivity of 67%, and specificity of 77% (Bradbury et al., 2009).

When radiographic masses are detected in the caudal abdomen, retrograde urethrography and positive contrast cystography can be used to locate the bladder and prostate and to distinguish these organs from others; however, they do not provide specific information regarding the etiology of the diseases. Additionally, contrast radiographs (Figure 1) can be employed to determine whether cystic structures are located within the prostate or in the periprostatic region (Assis et al., 2015).

Contrast medium extravasation into the prostate is nonspecific and may occur under normal conditions. However, in the presence of lesions communicating with the urethra, contrast leakage can appear as large, irregular cavitations of contrast accumulation with either rough or smooth margins, potentially indicating neoplastic processes. The absence of contrast extravasation does not exclude prostatic abnormalities. Contrast filling defects at the proximal end of the urethra may be associated with transitional cell carcinoma (Johnston et al., 1991).

Radiography is seldom used for the evaluation of intrascrotal diseases due to the testes, epididymides, and scrotum having radiographic densities similar to those of the surrounding soft tissues (Johnston et al., 1991). Additionally, scrotal enlargement may be observed; however, it is not possible to distinguish between a testicular mass, intrascrotal fluid, or scrotal thickening (Russo et al., 2021).

The possibility of a neoplastic ectopic testis should be considered when radiographic evaluation reveals abdominal masses in animals with no history of castration and in which testicular absence in the scrotum is confirmed by palpation. For radiographic characterization, the neoplastic abdominal testis must be sufficiently enlarged. When visible radiographically, it is most commonly located between the kidney and the inguinal canal, exhibiting soft tissue opacity. Identification and displacement of organs adjacent to the neoplastic abdominal testis should be assessed using both lateral and ventrodorsal projections in order to exclude other potential anatomical sites of origin. Differentiating a testicular neoplasm from masses originating in other abdominal locations can be challenging on radiographs. In some cases, the neoplastic testis may be visualized in the subcutaneous tissue of the caudoventral inguinal region of the abdomen, typically appearing as a soft tissue opacity mass (Birchard and Nappier, 2008; Assis et al., 2015).

Penile neoplasms are relatively rare in dogs (Hall et al., 1976). For penile evaluation, radiographic findings such as soft tissue swelling and osteolysis of the baculum have been reported (Burchell et al., 2014). However, these findings are not specific to any particular tumor type, thus requiring histopathological analysis to determine the nature of the lesion, as previously described in cases of osteosarcoma (Peppler et al., 2009) and hemangiosarcoma of the baculum (Burchell et al., 2014).

**Figure 3:**
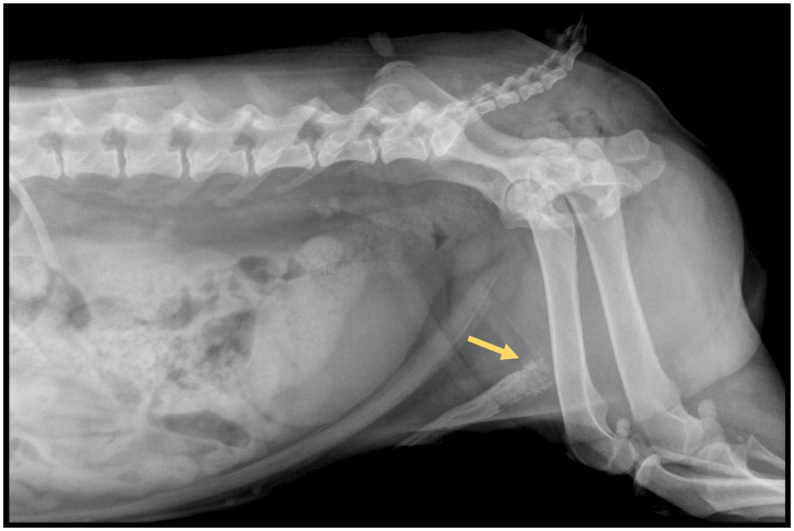
Lateral radiographic projection of a dog with neoplasia in the os penis. An irregular, mineral radiopaque enlargement is observed adjacent to the proximal portion of the os penis (arrow). Image kindly provided by Luciana Burguese – PROVET.

Radiography has also allowed the identification of changes such as urethral obstruction and penile edema secondary to soft tissue enlargement, as described in a case histopathologically confirmed as penile lymphosarcoma (Michels et al., 2001). It is important to highlight that the combination of imaging findings, including ultrasonography, plain radiography, and contrast studies, with histological evaluation is essential for the diagnosis and characterization of penile neoplasms (Figure 3).

## Applicability of ultrasonography

**Figure 4:**
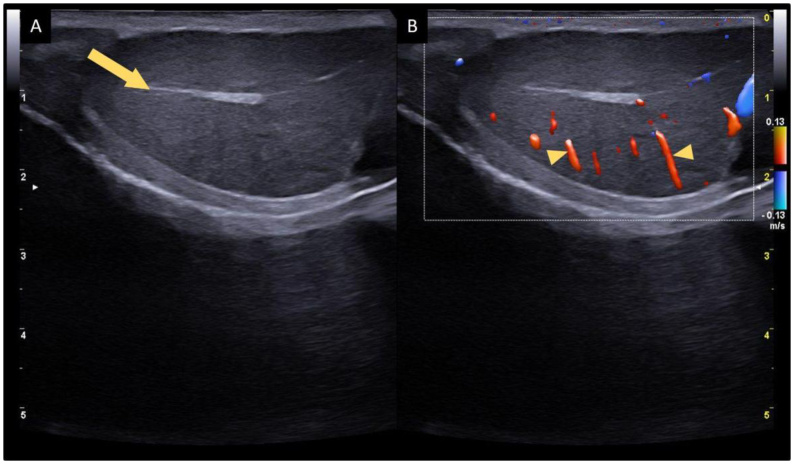
Ultrasonographic images of a normal canine testicle in the sagittal plane. (A) B-mode evaluation of the homogeneous parenchyma, well defined margins, regular contours and an echogenic mediastinal line (arrow); (B) Color Doppler evaluation of the testicular vascularization with normal distribution of the intratesticular branches (arrowheads) of the testicular artery.

**Figure 5:**
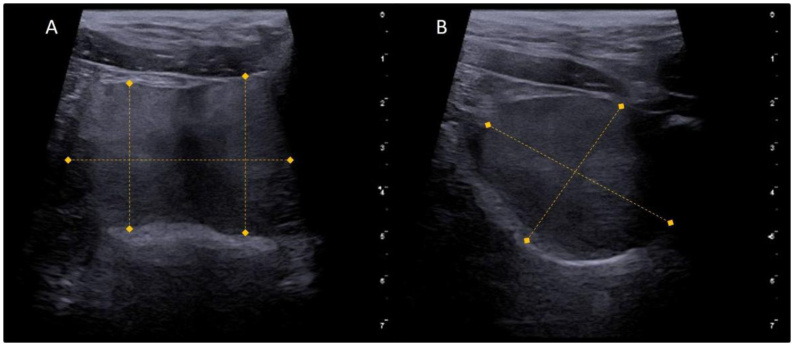
Ultrasonographic images of a normal canine prostate in transverse (A) and longitudinal (B) sections, showing the measurements taken during its evaluation.

Ultrasonography is the imaging modality of choice for evaluating the male reproductive tract in dogs and cats, as it allows for the assessment of organ size and parenchymal structure (Figures 4 and 5), as well as guidance for sample collection procedures such as fine-needle aspiration (FNA) or tissue and lesion biopsy (Mantziaras, 2020; Meomartino et al., 2021). B-mode ultrasonography is an effective tool for detecting morphological alterations; however, it presents limitations in evaluating functional aspects and distinguishing between benign and malignant lesions (Volta et al., 2014). In this context, the use of multiparametric approaches, combining different ultrasonographic techniques, has emerged as a promising strategy to improve diagnostic accuracy in the assessment of reproductive health (Mantziaras and Luvoni, 2020).

**Figure 6:**
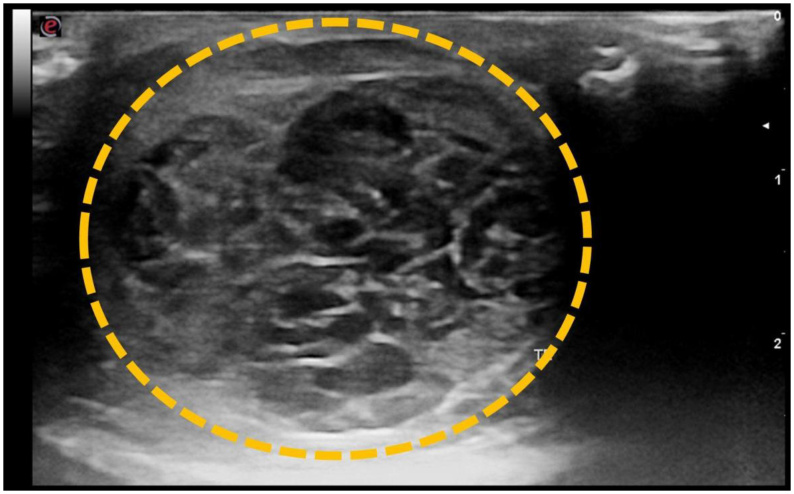
Ultrasonographic image of intra-abdominal testicular neoplasm in a dog. Mixed testicular tumor (seminoma and Sertoli cell tumor), characterized by a poorly defined, heterogeneous, cavitary mass occupying nearly the entire testicular parenchyma (dashed line).

**Figure 7:**
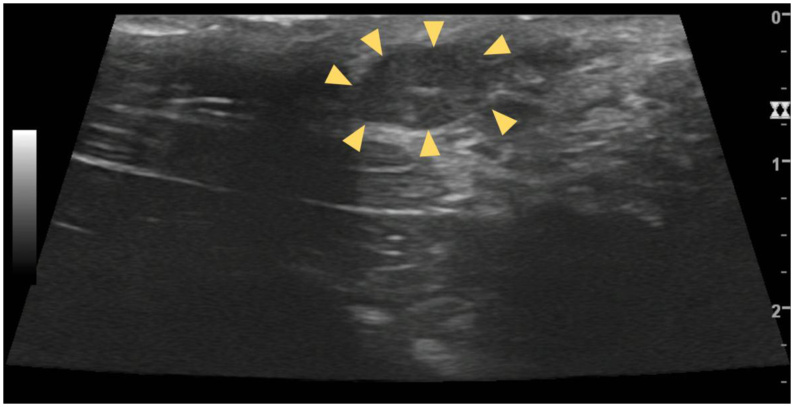
B-mode ultrasonography of an inguinal ectopic testicle in the cat, in longitudinal view. Arrowheads point to the hypoplastic or atrophic testicle with a hypoechoic parenchyma and ill-defined mediastinal line. Malignancy is among the differentials for this case.

Testicular tumors, illustrated in Figure 6, are common in dogs, particularly in older individuals, with reported prevalence ranging from 2% to 60% (McEntee, 2002; Grieco et al., 2008; Orlandi et al., 2022). Ultrasonography enables the detection of focal lesions suggestive of neoplastic involvement or potential testicular torsion (Yates et al., 2003). Sertoli cell tumors are most frequently associated with retained testes, followed by seminomas (England, 1995; Mostachio et al., 2007; Foster, 2012; Bertoldi et al., 2014; Stokowski et al., 2016). Furthermore, the testis may also serve as a site of metastatic spread from other primary neoplasms (Lucas et al., 2012). Testicular tumors are rare in cats (Figure 7); however, interstitial cell tumors, teratomas, Sertoli cell tumors, and seminomas have been reported in this species (McEntee, 2002; Miller et al., 2007).

**Figure 8:**
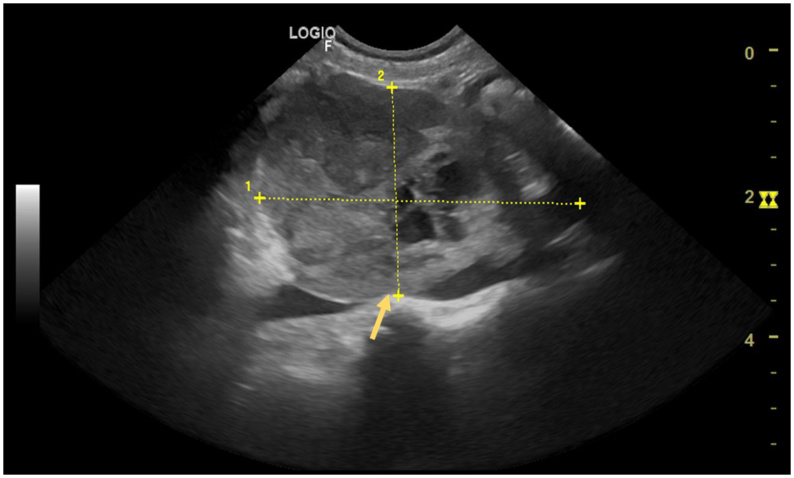
Ultrasonographic image of a Sertoli cell tumor in an intra-abdominal ectopic testis causing mechanical compression of the caudal vena cava. A well defined, heterogeneous, solid-appearing mass with interspersed cavitary areas is observed overlapping the caudal vena cava, occluding its lumen (arrow).

B-mode ultrasonography is widely used for the detection of testicular tumors and allows for the differentiation between intratesticular and extratesticular conditions that lead to scrotal enlargement, as well as the identification of ectopic testes (Pugh and Konde, 1991; Gradil et al., 2007; Orlandi et al., 2022). The development of neoplasia and/or testicular torsion is also associated with cryptorchidism, as illustrated in Figure 8 (Yates et al., 2003). Testicular lesions are more commonly focal rather than diffuse, occupying only part of the testicular parenchyma, and typically present as hypoechoic areas without specific sonographic features that allow differentiation based on tumor type (Orlandi et al., 2022). Additionally, a heterogeneous echotexture may be observed, with or without loss of definition of the mediastinum testis. Hemorrhage and necrosis may also be present, appearing as disorganized hyperechoic or hypoechoic regions. These tumors can cause generalized testicular enlargement, obliteration of the testicular mediastinum and epididymis, and distortion of the normal organ anatomy (Assis et al., 2015).

Although B-mode ultrasonography allows for detailed visualization of the testicular architecture and parenchyma, it does not permit accurate differentiation among Sertoli cell tumors, seminomas, interstitial cell carcinomas, abscesses, or hematomas (Mantziaras, 2020).

The scrotum is a musculocutaneous pouch that houses the testes, epididymides, and deferent ducts. Any tumor involving the skin may potentially affect the scrotum in both dogs and cats (McEntee, 2002). Neoplastic lesions of the scrotum may account for approximately 0.4% of all cutaneous neoplasms (Trappler et al., 2014).

The prostate is the only accessory sex gland in dogs, completely encircling the proximal urethra at the level of the bladder neck and is bilobed in structure. In cats, the prostate has been described as a “rudimentary” organ, presenting as a compact structure composed of tubular glandular epithelium, symmetrically distributed dorsolaterally to the urethra, extending from its cranial portion caudodorsally to the cranial border of the pelvic symphysis. Bulbourethral glands are present in all domestic mammals, with the exception of dogs (Mattoon and Nyland, 2002; Konig and Leibich, 2016).

Although ultrasonography is the imaging modality of choice for evaluating the canine prostate, sonographic findings of prostatic neoplasms are nonspecific and do not allow for differentiation among tumor types (Mantziaras, 2020). In felines, ultrasonographic assessment of the prostate and bulbourethral glands has been scarcely described in the literature (Dimitrov et al., 2010).

Prostatic neoplasms and BPH may initially present with similar ultrasonographic parenchymal features. In advanced stages of neoplasia, the parenchyma may appear heterogeneous, hyperechoic or mixed, with irregular anechoic regions and areas of calcification (Assis et al., 2015). Ultrasonographic evaluation of the medial iliac lymph node may be useful, as enlargement, hypoechogenicity, and possibly increased vascularity can be observed in cases of suspected prostatic inflammation or neoplasia (Mantziaras, 2020). Similar to radiography, the detection of prostatic mineralization by ultrasonography in neutered dogs has a 100% positive predictive value for malignancy (Bradbury et al., 2009).

Malignant lymphoma of the canine prostate is rare and has been reported primarily as a manifestation of multicentric disease rather than as a primary prostatic lymphoma. This type of neoplasm can exhibit a wide variety of ultrasonographic appearances. However, a predominance of hypoechoic lesions, whether diffuse, focal, or multifocal, is notable. Other common features include rounded or irregular margins, absence of mineralization, and ultrasonographic evidence of involvement of multiple organs, with or without lymphadenomegaly. Prostatomegaly was subjectively recorded in all patients, although it was not quantified through objective measurements (Di Donato et al., 2019).

### Doppler

Doppler ultrasonography has emerged as an advanced imaging modality for evaluating the reproductive tract of small animals, serving as a useful tool in the assessment of the prostate and testicles, including its applicability as a marker of semen quality and in the differentiation between benign and malignant lesions (Bigliardi et al., 2019; Mantziaras and Luvoni, 2020). However, the various tumor types are not distinguishable using this technique (Bigliardi et al., 2019). Doppler modalities such as color and pulsed Doppler assess the presence and direction of blood flow, as well as provide information on hemodynamic parameters, respectively (Orlandi et al., 2022).

**Figure 9:**
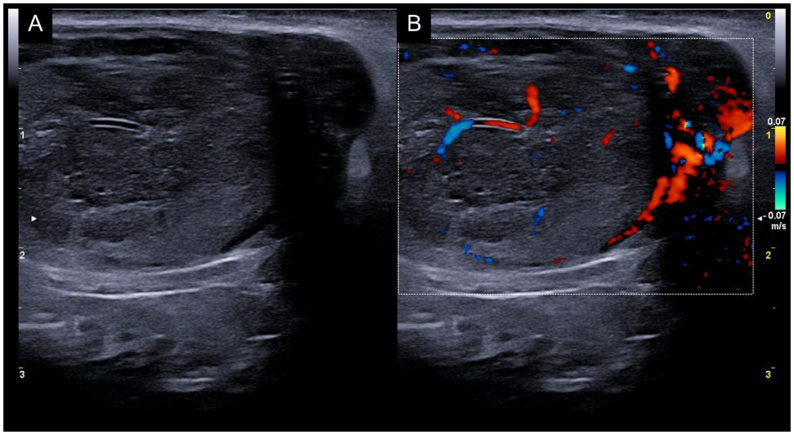
Ectopic testicle identified in the subcutaneous abdominal region of a 13-year-old Yorkshire dog with unilateral cryptorchidism. (A) Neoplastic findings, evaluated by B-mode ultrasound in the sagittal plane, showing heterogeneous parenchyma, increased dimensions, and evident abnormal vascular walls; (B) Color Doppler shows evident perilesional vascularization and deficient vascularization in the rest of the parenchyma.

In the evaluation of testicular tumors in dogs using color Doppler imaging, neoplastic lesions (Figure 9) demonstrate increased internal and peripheral blood flow when compared to inflammatory and degenerative lesions. An increased vascular index has been detected in solid tumors, while no blood flow was observed around cysts (Bigliardi et al., 2019).

Additionally, subtle variations in the tumor vascularization pattern may aid in their differentiation. Sertoliomas exhibited higher pulsatility and resistance indices in the testicular and pampiniform arteries, whereas Leydig cell tumors showed a predominantly peri- and/or intralesional blood flow pattern (Orlandi et al., 2022).

Although the vascular patterns of testicular blood flow in adult cats have already been established (Brito et al., 2015), the literature remains scarce regarding species-specific abnormalities (Trautwein et al., 2024).

Even though adenocarcinomas cause changes in prostatic shape, increased volume, loss of capsular integrity, and heterogeneous echogenicity, with anechoic, mineralized, and nodular areas, Doppler ultrasonography has limitations in differentiating benign from malignant prostatic diseases (Troisi et al., 2015; Mantziaras, 2020).

### Elastography

Elastography is a recent ultrasonographic technique that allows for both qualitative and quantitative assessment of tissue stiffness, based on two main categories: strain elastography (which requires external mechanical compression and assesses tissue elasticity) and shear wave elastography (Zappone et al., 2024). Over the past decade, it has been applied to the evaluation of the male reproductive system in dogs and cats, in association with B-mode ultrasonography (Brito et al., 2015; Feliciano et al., 2015).

While this examination is valuable for assessing tissue health, its application presents certain limitations, as lesions of inflammatory or neoplastic origin may alter histological composition in various ways. Nonetheless, the availability of reference values for evaluating structures of the reproductive tract in small animals can support the rapid and non-invasive differentiation between benign and malignant conditions (Feliciano et al., 2015; Zappone et al., 2024). Furthermore, as an ultrasonographic technique, it is frequently integrated into multiparametric assessments, thereby contributing to a more accurate diagnosis (Mantziaras and Luvoni, 2020; Cantisani et al., 2021).

**Figure 10:**
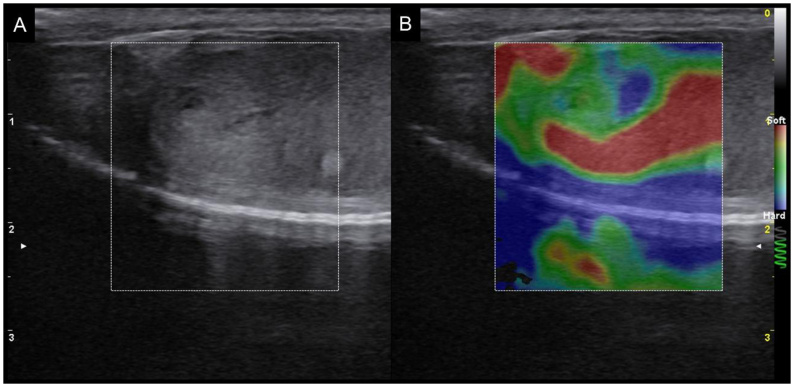
Qualitative elastography of a testicular Sertolioma. (A) B-mode image showing a coarse-textured, mildly nodular region with a small, poorly defined hypoechoic structure interspersed within the testicular parenchyma; (B) The color map reveals a central area of the lesion with regions of increased stiffness (blue), intermediate stiffness (green), surrounding testicular parenchyma with predominantly lower stiffness (red), and a capsular region with increased stiffness (blue). The elastogram also provides improved delineation between the nodular region and adjacent tissue. Image kindly provided by Marjury Cristina Maronezi.

**Figure 11:**
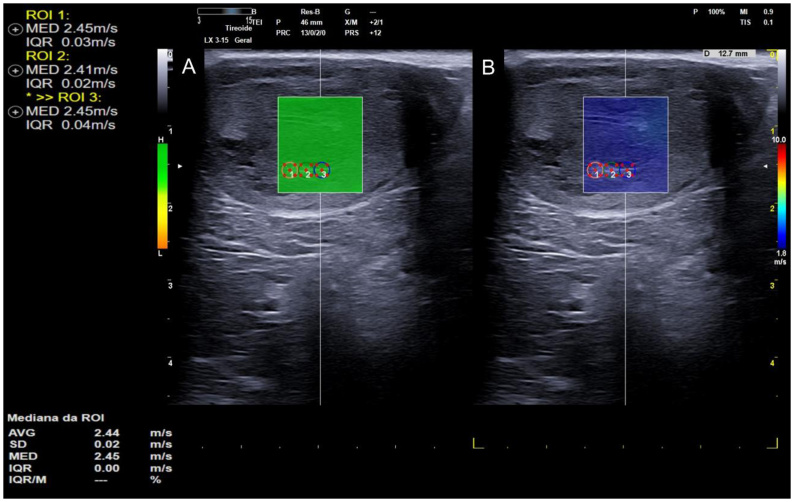
Quantitative shear wave elastography of a neoplastic ectopic testicle (sertolioma) from the same dog shown in Figure 9. (A) The green box shows the map with high-quality sample acquisition according to the scale bar on the left side (green, high quality; yellow and orange, low quality); (B) The blue box shows the elastogram (red, stiff; blue, soft) with three circular ROIs (1, 2, and 3) for acquiring stiffness values in m/s.

Elastography enhances the diagnosis of testicular neoplasms due to its ability to distinguish between healthy and diseased testes based on tissue stiffness (Zappone et al., 2024), as demonstrated in Figures 10 and 11. This technique is already applied in human medicine for differentiating neoplastic lesions, as well as characterizing malignancy in testicular lesions (Cantisani et al., 2021), showing 100% sensitivity for the detection of testicular tumors, with observed increases in testicular parenchymal stiffness (Aigner et al., 2012).

Increased stiffness associated with ultrasonographic findings such as heterogeneity allows for the identification of abnormalities, although these are insufficient for differentiating the type of testicular lesion in dogs (Feliciano et al., 2016). As described in humans, neoplastic testicular lesions in dogs tend to exhibit greater stiffness than non-neoplastic lesions (Glińska-Suchocka et al., 2014). However, tissue stiffness values do not differ among the various types of testicular neoplasms presented (Zappone et al., 2024).

The literature regarding elastographic parameters of diseased prostatic tissue in dogs remains limited (Cintra et al., 2020; Mantziaras and Luvoni, 2020), and to date, no reference values exist for elastographic parameters in cats. Nevertheless, this ultrasonographic technique has been used as an adjunct tool in the diagnosis of prostatic neoplasms in humans (D’Anastasi et al., 2011); similarly to testicular tissue, increased stiffness is observed when compared to the parenchyma of healthy glands or those with benign lesions (Ophir et al., 2002). Likewise, recent studies in dogs have demonstrated increased stiffness in the parenchyma of diseased prostates (Domosławska et al., 2018; Bucci et al., 2023).

### Contrast-enhanced ultrasonography (CEUS)

Contrast-enhanced ultrasonography (CEUS) is a technique based on the administration of a contrast agent composed of gas microbubbles encapsulated by a phospholipid membrane, which amplifies the ultrasound signal and has been used to evaluate the male reproductive tract in small animals (Quaia, 2005; Mantziaras, 2020). This imaging modality enables the study of tissue microvascularization and contributes to better identification and characterization of perfusion in lesions and inflammatory processes affecting reproductive organs such as the testicles and prostate, thereby improving diagnostic accuracy when used in conjunction with conventional ultrasonography (Volta et al., 2014; Orlandi et al., 2022).

In addition to being a real-time ultrasonographic technique that is safe, with minimal contraindications and side effects, the most recent contrast agents used in this method, such as sulfur hexafluoride, offer prolonged stability, do not extravasate into the extracellular space, and allow both qualitative and quantitative assessment of normal and altered organ perfusion (Ohlerth and O’Brien, 2007; Haers and Saunders, 2009; Pettina et al., 2024).

**Figure 12:**
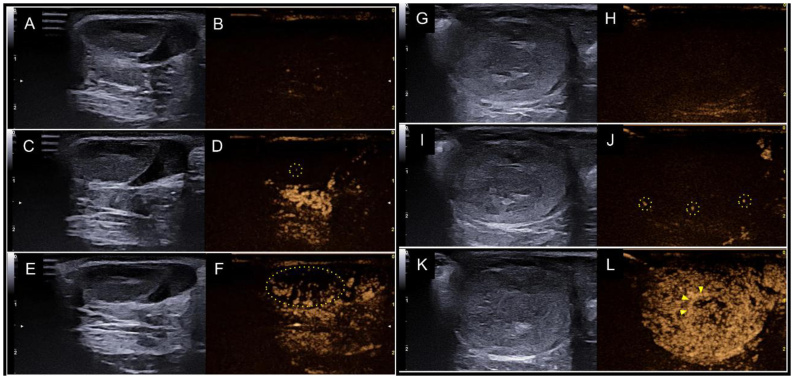
B-mode ultrasound and CEUS images of a 13-year-old Yorkshire dog with unilateral cryptorchidism. (A), (C), and (E): testicle located in the scrotal sac, evaluated by B-mode ultrasound in the sagittal plane, showing hypoechoic parenchyma and reduced dimensions. (B), (D) and (F): the same testicle evaluated by CEUS at different contrast phases: immediately after contrast administration (0s) (B); late wash-in (15s) and the moment of detection of microbubbles (dotted lines) in the hypoplastic testicular parenchyma (D); and late peak enhancement (31s) homogeneous, with poor identification of intratesticular branches of the testicular artery in the dorsal portion of the testicle (dotted lines) (F). (G), (I) and (K): ectopic testicle, identified in the subcutaneous region of the abdomen of the same animal, with neoplastic findings (sertolioma), evaluated by B-mode ultrasound in the sagittal plane, showing heterogeneous parenchyma and increased dimensions. (H), (J) and (L): the same testicle evaluated by CEUS at different contrast phases: immediately after contrast administration (0s) (H); early wash-in (8s) and the moment of microbubble detection (dotted lines) in the neoplastic testicular parenchyma (J); and peak enhancement (16s) with early distribution of contrast throughout the parenchyma, showing a nodular lesion, perilesional vessels (arrowheads) and intralesional vessels (L).

Non-neoplastic lesions exhibited mild to moderate homogeneous enhancement, with well defined margins during the wash-in phase (Figures 12D and 12F), which remained consistent during the wash-out phase (Volta et al., 2014). In contrast, neoplastic and inflammatory testicular lesions demonstrated rapid wash-in, prolonged enhancement duration (Figures 12J and 12L), and delayed wash-out. Perfusion parameters were higher in neoplastic lesions, with hyperenhancement being strongly associated with malignancy, showing a sensitivity of 87% and specificity of 100% (Russo et al., 2021). Lesions with persistent internal vascularization and hypoechoic to isoechoic parenchyma were associated with seminomas. Degenerated and atrophic testes exhibited poor contrast enhancement, lower than that of normal tissue (Volta et al., 2014).

One study reported difficulty in differentiating tumor types in testicular lesions based on enhancement patterns alone; however, CEUS outperformed color Doppler, power Doppler, and B-flow by detecting a tumor lesion that these other modalities, including B-mode, failed to identify (Orlandi et al.,2022). Similarly, another study reported that CEUS can differentiate testicular abnormalities and neoplastic lesions, but cannot characterize tumor types (Sinagra et a., 2023).

To date, CEUS has only been used to assess feline testes for the establishment of normal reference parameters (Brito et al., 2015); however, it holds potential as a diagnostic tool to improve ultrasonographic accuracy in identifying benign and malignant conditions affecting this organ, thus supporting earlier clinical and surgical decision-making.

Canine prostatic neoplasms affect elderly individuals, both neutered and intact (Krook, 1954). In cats, these tumors are extremely rare; however, as in dogs, they are typically diagnosed at an advanced stage of the disease, with a poor prognosis due to their aggressiveness and metastatic potential (Caney et al., 1998; Oliveira et al., 2019; Palmieri et al., 2022).

The main challenge in diagnosing prostatic disorders, particularly neoplasms, lies in the limited ability to detect them early and to differentiate benign from malignant processes, due to the overlapping features observed on imaging studies, especially with conventional ultrasonography (Mantziaras, 2020; Russo et al., 2021; Pettina et al., 2024).

Early-stage prostatic neoplasms can be identified through CEUS due to increased blood flow resulting from tumor-induced neoangiogenesis. This is evidenced by rapid asymmetric inflow, increased focal enhancement, and visualization of tortuous intraprostatic vessels, making CEUS the most effective technique when compared to color and power Doppler (Vignoli et al., 2011; Spada et al., 2025).

Specific perfusion patterns have been identified in canine prostates with and without prostatic disease. Time to peak (TTP; seconds) and mean transit time (MTT; seconds) were shorter in prostates affected by pathology; however, no significant differences were observed among the conditions (BPH, prostatitis, and tumors) due to technique-related variables and the lack of well established protocols for each organ, such as patient preparation and ultrasound setting adjustments. In this study, qualitative assessment of diseased prostates allowed differentiation from healthy prostates by the absence of the characteristic centripetal perfusion pattern and the loss of subcapsular arterioles. Furthermore, it was possible to identify distinct vascular patterns associated with conditions such as BPH, prostatitis, adenocarcinoma, and lymphoma (Troisi et al., 2015).

CEUS stands out for its excellent ability to characterize blood perfusion and to detect prostatic lesions that are often missed by conventional methods. For this reason, it represents an advanced and promising imaging tool for early diagnosis, allowing for differentiation between benign and malignant processes. In addition, CEUS is useful for monitoring the regression of these conditions following treatment, as well as for guiding targeted biopsies and radiofrequency ablation procedures, thereby contributing to enhanced diagnostic and therapeutic accuracy (Liu et al., 2008; Russo et al., 2021; Sinagra et al., 2023).

## Multiparametric ultrasound (MPUS)

The combination of different imaging techniques for detecting abnormalities in the reproductive system of small animals is already a reality. Although definitive diagnosis is confirmed by cytology or histopathology, the investigation of neoplasms in the reproductive structures of dogs and cats often integrates information obtained from radiographic and ultrasonographic findings, aiming to achieve a more accurate prognosis regarding lesion involvement, adjacent structures, and potential metastatic sites (Michels et al., 2001; Peppler et al., 2009; Burchell et al., 2014).

Due to recent and promising advances in various ultrasonographic modalities, the term *Multiparametric Ultrasound (MPUS) Imaging* has emerged as a comprehensive approach that combines multiple ultrasonographic techniques such as B-mode, Doppler, elastography, and CEUS (Sidhu, 2015). This has been made possible by technological advancements in imaging equipment alongside the development of novel advanced ultrasound techniques. These methods were not designed to be used in isolation but rather to enhance the diagnostic sensitivity of conventional B-mode ultrasonography (Mantziaras and Luvoni, 2020).

Currently, multiparametric assessment stands out as the most accurate diagnostic method for ultrasonographic evaluation of reproductive health in both humans and small animals (Mantziaras and Luvoni, 2020). It has proven highly valuable in detecting neoplastic lesions and assessing malignancy. In human medicine, it is already used in the evaluation of prostate (Kaneko et al., 2022) and testicular cancer (Cantisani et al., 2021); and studies are underway to establish veterinary applications, including protocols for evaluating reproductive organs in male dogs and cats (Brito et al., 2015; Mantziaras and Luvoni, 2020; Aires et al., 2025).

## Conclusion

Imaging methods such as radiography and ultrasonography stand out for being low-cost, accessible, and safe for the patient. Although they have certain limitations, these modalities are important tools for screening and monitoring disorders of the male reproductive system in dogs and cats. The application of advanced ultrasonographic modalities and the multiparametric approach provide valuable diagnostic support, increasing accuracy in the detection of neoplastic lesions and the identification of malignancy-related features in these animals. However, it is important to note that despite their relevance, these methods do not replace cytological or histological examinations, which remain the gold standard for the confirmation and differentiation of benign and malignant neoplasms in the reproductive system of dogs and cats.

## Data Availability

No research data was used.
